# Acquisition of Chinese characters: the effects of character properties and individual differences among second language learners

**DOI:** 10.3389/fpsyg.2015.00986

**Published:** 2015-08-03

**Authors:** Li-Jen Kuo, Tae-Jin Kim, Xinyuan Yang, Huiwen Li, Yan Liu, Haixia Wang, Jeong Hyun Park, Ying Li

**Affiliations:** ^1^Department of Teaching, Learning, and Culture, Texas A&M UniversityCollege Station, TX, USA; ^2^Independent ResearcherTaipei, Taiwan; ^3^Chinese Studies, Department of Modern Languages, Carnegie Mellon UniversityPittsburg, PA, USA; ^4^Department of East Asian and Middle East Studies, Duke UniversityDurham, NC, USA; ^5^Confucius Institute, University of PittsburghPittsburgh, PA, USA; ^6^Independent ResearcherBeijing, China

**Keywords:** Chinese character, orthography, second language learning, character acquisition, learner difference

## Abstract

In light of the dramatic growth of Chinese learners worldwide and a need for cross-linguistic research on Chinese literacy development, this study drew upon theories of visual complexity effect (Su and Samuels, 2010) and dual-coding processing (Sadoski and Paivio, 2013) and investigated (a) the effects of character properties (i.e., visual complexity and radical presence) on character acquisition and (b) the relationship between individual learner differences in radical awareness and character acquisition. Participants included adolescent English-speaking beginning learners of Chinese in the U.S. Following Kuo et al. (2014), a novel character acquisition task was used to investigate the process of acquiring the meaning of new characters. Results showed that (a) characters with radicals and with less visual complexity were easier to acquire than characters without radicals and with greater visual complexity; and (b) individual differences in radical awareness were associated with the acquisition of all types of characters, but the association was more pronounced with the acquisition of characters with radicals. Theoretical and practical implications of the findings were discussed.

## Introduction

Acquisition of the relationship between the written form of a word and its meaning has been a central topic in the field of literacy development (Kuo and Anderson, 2006). Multiple theories have been proposed to account for the acquisition of reading skills (Kuo and Anderson, 2008; Alvermann et al., 2013). However, few have concurrently examined the properties of the acquired words and the individual differences of the learners. The present study aims to fill this gap with a focus on the acquisition of Chinese characters among non-native adolescent beginning learners of Chinese.

Research on Chinese literacy acquisition has increased dramatically over the past two decades (Shu and Anderson, 1997; Nagy et al., 2002; Packard et al., 2006; Wu et al., 2009). Compared with alphabetic languages, which are more widely studied, the Chinese writing system is substantially different in terms of visual configuration and the correspondences among sound, meaning, and graphemes. Over the past two decades, there has been a shift in the focus of research on Chinese literacy. Previously, studies on reading in Chinese primarily examined the processing and acquisition of characters in terms of its visual complexity (i.e., the number of strokes within a character) or radical presence (i.e., the presence of the stroke patterns that represent the general semantic category of a character) (Shu and Anderson, 1997; Perfetti and Tan, 1998; Feldman and Siok, 1999; Zhou et al., 1999). Recently, however, research has focused more on the influences of individual difference in visual skills (e.g., McBride-Chang et al., 2005b; Tong et al., 2009; Luo et al., 2013) or radical awareness (i.e., understanding of stroke patterns that represent the general semantic category of a character) (e.g., Ho et al., 2003a; Wu et al., 2009) on reading achievement.

The present study extends the existing research in two directions based on theories of visual complexity effect (Su and Samuels, 2010) and dual-coding processing (Sadoski and Paivio, 2013). First, two major character properties, visual complexity and radical presence, will be examined simultaneously. Second, individual learner difference in radical awareness is taken into account in this study as well as how it affects the acquisition of Chinese characters that vary in visual complexity and radical presence. By studying character properties and individual difference simultaneously, we aim to provide more comprehensive insights to literacy development in general as well as Chinese character acquisition.

The following sections will first present a comparison between the Chinese and the alphabetic language writing system. The next section will review theories of visual complexity and dual-coding processing. The subsequent sections will discuss the importance of character properties and individual learning difference in the acquisition of Chinese characters.

### The chinese writing system

The Chinese writing system is logographic in that each *character* represents one morpheme instead of an individual phoneme of the spoken language (Shu and Anderson, 1997; Whitney, 1998; Feldman and Siok, 1999). This is largely different from alphabetic-phonemic languages where word recognition is letter-recognition based. Over 80% of the modern Chinese characters are compound characters composed of a *semantic radical* and a *phonetic component* (Hoosain, 1991; Chen et al., 1996; Williams and Bever, 2010). The semantic radical indicates the meaning of a character, and the phonetic component indicates the pronunciation of that character (Hoosain, 1991; Chen et al., 1996). Some radicals can be independent characters and stand individually while others are dependent and can only occur within or together with other characters. For instance, the semantic radical 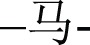 *(horse)* in the character 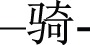 *(ride)* is an independent character itself, whereas the radical 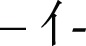 *(person)* in the character 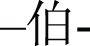 *(uncle)* had to be combined with other components to from a character. Except for a few instances, the position of the same radical is generally consistent in characters, and most semantic radicals are either in the left or on the top of a character.

A large number of Chinese characters share the same radicals, and these characters are usually related in meaning and fall into the same semantic category (Tong et al., 2009). For example, the characters 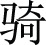 (ride), 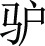 (donkey), and 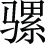 (mule) share the same radical 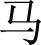 (horse), which represents *horse*. The semantic feature shared by these characters is fairly obvious, and thus these characters are called radical-transparent characters. There are also small groups of radical-opaque characters, which share the same sematic radicals but are not semantically related (T'sou, 1981; Flores D'arcais, 1992). For example, the character for the word *swallow*, 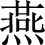, does not contain the radical 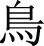, which is present in most of the Chinese characters that represent different species of birds (Shu and Anderson, 1997). Instead, the radical for 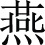
is “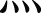,” which represents the *fire* category and has nothing to do with birds. Nevertheless, approximately 70% of the Chinese characters taught to beginning learners are radical-transparent (Shu et al., 2003). Therefore, in general, the semantic category of the characters can be inferred from their radicals in early Chinese literacy development.

Chinese and alphabetic languages are also contrasted in other aspects: (a) the association between the semantic information and the phonetic units (b) the correspondence between the phonetic units and the graphemes. First, Chinese characters are monosyllabic, that is, each character represents one syllable. In addition, Chinese is a tonal language so a change in the pitch of a vowel sound of a syllable can change the meaning of the syllable. For instance, the syllable /ma/ can have different meanings when it is associated with different tones, as in /ma1/, with a flat tone, for *mother*, /ma2/, with a rising tone, for *numb*, /ma3/, with an inflected tone, for *horse*, and /ma4/, with a falling tone, for *scold*.

Second, Chinese has a large number of *homophones*, words with the same sound but different meanings. For instance, these characters, 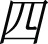 *(four)*, 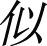 *(similar)*, 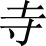 *(temple)*, all share the same syllable and tone, */si4/* (falling tone), but each is represented in a different character with a different meaning. In other words, the same syllable with the same tone can be represented by distinct characters and have completely different meanings. It is estimated that on average each Chinese syllable has five homophones (McBride-Chang and Zhong, 2003). Contrastively, English has a much smaller number of homophones. The prevalence of homophones in Chinese adds to the complexity of vocabulary acquisition in Chinese. Hence, radicals become even more critical in literacy development because of the semantic clues they provide for homophonic characters.

### Dual-coding theory, radical presence, and character processing

Dual-Coding Theory (Paivio, 1971) postulates that mental representation comprised two distinct codes or systems: the *verbal* code and the *non-verbal* code. The verbal code represents and processes linguistic information whereas the non-verbal code represents and processes non-linguistic objects and events. The Dual-Coding Theory, originally a theory developed for general cognition, has recently been adopted to account for reading processes (Sadoski and Paivio, 2013). According to Sadoski and Paivio (2013), reading involves three distinct dimensions of processing: (a) representational processing, (b) associative processing, and (c) referential processing. During representational processing, visual input is initially activated (e.g., recognizing familiar words or parts of words), but semantics may or may not be involved. In contrast, associative and referential processing always activate meaningful comprehension. Associative processing refers to the spreading of the initial activation *within* a verbal code that is often related to meaningful comprehension. For example, the word *cake* may be associated with verbal activation including *sweet, bakery, birthday*, and *candles*. On the other hand, referential processing refers to the spreading of the initial activation *between* codes that involves meaningful comprehension. For example, the word *cake* may be associated with non-verbal activation including mental images of *a cake displayed at a bakery* or *a birthday cake with candles*. Such mental images may be further activated referentially to other words in the system.

Dual-Coding Theory has important implications for reading in Chinese because radical evokes verbal and non-verbal activations quite differently from patterns in alphabetic languages. Other things being equal, Dual-Coding Theory assumes that Chinese characters with a radical are more likely to evoke verbal and non-verbal activation compared to the characters without radicals. In other words, the presence of radicals may facilitate character processing and acquisition (Sadoski and Paivio, 2013; Kuo et al., 2014). Furthermore, Sadoski and Paivio (2013) also proposed that individual differences in visual skills and linguistic knowledge may also contribute to the three distinct dimensions of processing (i.e., representational, associative, and referential processing).

In accordance with predictions derived from Dual-Coding Theory, research has demonstrated that readers of Chinese process radicals in an automatic manner. There are two main facets of radical information that readers need to attend to: semantics and positional regularity. Regarding semantics, previous research has shown that both Chinese-speaking adults (Zhang et al., 1990; Miao and Sang, 1991; Taft and Zhu, 1994, 1997; Chen et al., 1996; Feldman and Siok, 1999; Taft et al., 1999) and children (Shu and Anderson, 1997; Ho et al., 1999; Wu et al., 2009) tend to activate the meanings of radicals while they recognize characters, and such a tendency is more noticeable when processing low-frequency characters than high-frequency characters (Shu and Zhang, 1987; Miao and Sang, 1991; Shu and Anderson, 1997). Furthermore, activation of the meanings of radicals is more pronounced among older readers than younger ones (Shu and Anderson, 1997; Ho et al., 2003a). Although children as young as 6 years of age show a basic understanding of the semantic aspect of radicals, it is generally not until third grade when they become fully knowledgeable of a radical's functions (Shu and Anderson, 1997; Ho et al., 2003a; Luo et al., 2011). Researchers have also reported a substantial association between the understanding of the semantic functions of radicals and literacy development (Shu and Anderson, 1997; Ho et al., 2003a). For example, in Shu and Anderson (1997), a study with Chinese-speaking children from grades 1 to 3, it was found that a strong relationship existed between knowledge of the meanings of radicals and reading achievement. Proficient readers of Chinese distinguished themselves from poor readers in that they are better at decomposing new characters into radicals and using their knowledge of radicals to infer the meanings of new characters. Subsequent intervention studies confirmed that readers' knowledge of radicals is significantly related to several aspects of literacy skills, such as character writing and reading comprehension among beginning native Chinese-speaking readers in early elementary grades (Nagy et al., 2002; Wu et al., 2009).

Another important property of radical is positional regularity. Stroke patterns representing a radical can occur in more than one location within Chinese. However, only the stroke pattern located following the positional regularity contributes to semantics of a character. Take the stroke pattern of 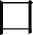 (with a meaning of mouth) as an example, it can be positioned in the left, right, top, or bottom part of a character as in 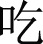 (eat), 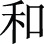 (and), 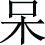 (dumb), and 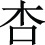 (almond), but only when 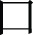 is located on the left side of the character, as in 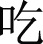 (eat), does 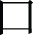 carries the meaning of mouth and contribute to the semantics of the character. Therefore, an understanding of the positional regularities of radicals, is fundamental in Chinese character processing because it contributes to accurate identification within a character and its semantic function. Understanding of the positional regularities of radicals develops from the grade 1 (Shu and Anderson, 1997; Chan and Nunes, 1998; Anderson et al., 2002), but is not fully mastered until grade 3 and beyond (Ho et al., 2003b; Liu et al., 2010).

### Theories of visual complexity effect and character processing

Visual complexity, another important property of Chinese characters, has also been extensively investigated (e.g., Su and Samuels, 2010). This line of research was originally motived by research on word-length effect in alphabetic languages (Just and Carpenter, 1987; Su and Samuels, 2010; Jalbert et al., 2011). The studies on the effects of word length on response latency have demonstrated that readers responded more slowly to words with longer letters through perceptual identification tasks (e.g., Aghababian and Nazir, 2000) or word-categorization tasks (e.g., Samuels et al., 1978; Su, 1997). Such word-length effect was found to be stronger in processing low-frequency words than high-frequency words (Ferrand, 2000; Juphard et al., 2004), and among younger readers than older readers (e.g., Samuels et al., 1978; Aghababian and Nazir, 2000; Bijeljac-Babic et al., 2004).

Would similar word-length effect also be observed in Chinese character processing? Earlier research demonstrated that both the number of strokes (e.g., Just and Carpenter, 1987; Tan and Peng, 1990) and the number of stroke patterns (e.g., Fang, 1994; Chen and Liu, 2000) could affect response latencies among adult readers of Chinese. More recently, Su and Samuels (2010) conducted a cross-sectional study to compare the effects of the numbers of strokes and stroke patterns among second and fourth graders and college students by using a character judgment task. It was found that character-complexity effect associated with the number of stroke patterns was not observed among any of the age groups, but character-complexity effect associated with the number of strokes was observed among the 2nd graders. These findings suggested that the number of strokes may serve as a more reliable indicator of character complexity than the number of stroke patterns. The finding that character-complexity effect is more prominent among younger than older readers concurs with research on the word-length effect with readers of alphabetic languages. Little research has been conducted to systematically examine how character complexity, indicated by the number of strokes, plays a role in the acquisition of new characters among beginning readers. To our knowledge, the only exception was Kuo et al. (2014), which was conducted with young beginning readers of Chinese who were native-speakers of the language. It was found that characters with less visual complexity were easier to acquire than characters with more visual complexity. No study has been conducted with older beginning readers who learn Chinese as a second language.

## The present study

As synthesized in the review in the previous sections, existing research on the acquisition of Chinese characters by beginning Chinese readers is limited in two respects. First, much of the research has focused on the relationships between either radical recognition and semantic awareness (e.g., Wu et al., 2009) or visual skills and character reading (e.g., McBride-Chang et al., 2005b) (for an exception, see Kuo et al., 2014). Drawing upon theories of visual complexity effect and dual-coding processing, the present study aimed to obtain a more comprehensive picture of Chinese literacy development by focusing on the *joint* effect of visual and semantic properties of the characters on the acquisition of the *meaning* of new characters as well as on how individual differences in radical awareness contribute to the acquisition process.

Second, the majority of the studies have been conducted with young native-Chinese speaking children. With the rapid economic growth in China and its expanding worldwide cultural influences, the number of Chinese learners has escalated worldwide. According to a 2012 report by Asian Society, between 2004 and 2008, the number of Chinese programs in elementary and secondary schools in the U.S. has increased by more than 200 percent from 263 to 779. Over 2000 high schools in the U.S. are offering Chinese as a foreign language. However, little research has been conducted with these learners of Chinese.

In sum, there are both theoretical and practical needs to conduct research on the role of character properties on Chinese character acquisition and the contribution of individual differences to this process among adolescent beginning learners of Chinese. In light of these gaps in the literature, the present study addressed the following two major research questions:

1. How do two core properties of Chinese characters, visual complexity and radical presence, affect the acquisition of Chinese characters among adolescent beginning Chinese readers? We hypothesize that the acquisition would be easier for characters with less visual complexity and with the presence of radicals.
2. How is radical awareness related to the acquisition of Chinese characters varying in visual complexity and radical presence? We hypothesize that radical awareness would be associated with the acquisition of Chinese characters with radicals but not characters without radicals, regardless of visual complexity.

## Methods

### Participants

Participants for the present study were 23 adolescent English-speaking learners of Chinese (12 females and 11 males) in grade 10 at a public high school located in a metropolitan area in a southern state in the U.S. By the time the study was conducted, the participants had taken beginning Chinese for 4 h a week for approximately 7 months as part of their foreign language requirement for graduation. According to the instructor, the majority of the participants reached a novice-high level in speaking and listening and a novice-mid level in reading and writing (American Council on the Teaching of Foreign Languages, 2012). None of the participants had documented learning disabilities. All participants and their parents completed consent forms reviewed and approved by the Institutional Review Board.

### Measures

The study included two measures: (1) character acquisition and (2) radical awareness. The character acquisition task attempted to investigate the role of visual complexity and radical presence in the acquisition of Chinese characters. The radical awareness measure intended to assess how individual differences may be related to acquisition of characters varying in radical presence and visual complexity.

#### Character acquisition task

Following Kuo et al. (2014) (see also Kuo, 2009; Kuo and Anderson, 2012; Kuo and Kim, 2014), the task involved two phases: *a study phase* and *a test phase*. Participants first learned a set of pseudo-characters during the *study phase*; in the following *test phase*, they were assessed on what they had studied during the study phase. Given the prevalence of homophones in Chinese, associating a new character with its meaning is more critical than with its pronunciation. Thus, the task focused on the association of the character and the meaning. Because previous research revealed that the effects of the two major character properties, radical presence and visual complexity, were particularly noticeable for characters with lower frequencies (Seidenberg, 1986; Shu and Zhang, 1987; Miao and Sang, 1991; Shu and Anderson, 1997), pseudo-characters instead of existing characters were used. By using pseudo-characters, the present study ensured that all stimuli were considered as unfamiliar, low frequency characters by the participants, which allowed the task to simulate the process of acquiring new characters.

##### Materials

Following Kuo et al. (2014), the experiment involved a 2 × 2 design with 48 pseudo-characters varying in their radical presence (i.e., with our without radicals within a pseudo-character) and visual complexity (i.e., with fewer than 8 strokes vs. with more than 10 strokes). The 48 pseudo characters were grouped into four conditions: (1) fewer-strokes, with radicals (FS-R); (2) more-strokes, with radicals (MS-R); (3) fewer-strokes, no radicals (FS-NR); and (4) more-strokes, no radicals (MS-NR). The pseudo-characters were composed with 12 semantic radicals and 12 simple characters. Based on findings from Su and Samuels (2010), visual complexity was indicated with the number of strokes rather than the number of stroke patterns. The average number of strokes in the fewer-stroke condition was 6.5 strokes (SD = 0.9, Min. = 5, Max. = 8) while the average number of strokes in the more-stroke condition was 11.3 strokes (SD = 0.9, Min. = 10, Max. = 13). The difference in the number of strokes between the two conditions was statistically significant, *t*_(46)_ = 17.19, *p* < 0.001. The ratio of the numbers of strokes in the fewer-stroke condition over the number of strokes in the more-stroke condition (0.42) was greater than the ratio of the number of strokes in simplified characters over the number of strokes in traditional characters (0.22); such difference has been shown to be sufficiently great enough in producing differences in visual processing (McBride-Chang et al., 2005a; for a review, see Li et al., 2010).

In the with-radical condition, a high-frequency semantic radical was combined with one or more simple characters to form a pseudo-character that looks like a real character in structure but does not exist in Chinese. Table 1 illustrates an example of the pseudo-character from each condition. For example, 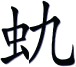 consisted of a semantic radical 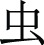
which typically means bug, and a simple character 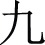 which has no association with the meaning or the sound of the pseudo-character. However, the pseudo- character 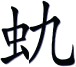 still looks legal in structure as the semantic radical 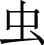 was located on the left side of the character as it is in real characters.

**Table 1:** **Sample pseudo-characters used in the four experimental conditions of the character acquisition task**.

	**With-radical (WR)**	**No-radical (NR)**
Fewer Strokes (FS)	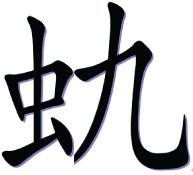	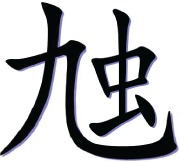
Number of strokes	8	8
Assigned meaning	A kind of worm	Mountain peak
More Strokes (MS)	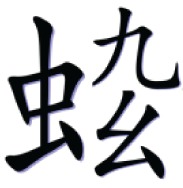	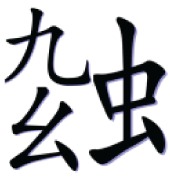
Number of strokes	11	11
Assigned meaning	A kind of bug	A kind of dance

In the without-radical condition, a high-frequency semantic radical was combined with one or more simple character but the semantic radical was not positioned on the left. For instance, 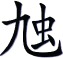 was considered a without-radical character because the semantic radical 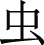 was located on the right side of the character and did not contribute to the meaning of the character.

Radicals and simple characters were counter-balanced across the conditions to ensure that each radical appeared once in each of the four conditions (i.e., FS-R, FS-NR, MS-R, MS-NR). Simple characters were used more in the two conditions with characters that had more strokes than in the other two conditions with characters that had fewer strokes. However, the frequencies of the occurrences of each simple character were the same across the more-stroke conditions and fewer-stroke conditions, respectively.

Since, none of the pseudo-characters existed in Chinese, meanings for them were randomly assigned for the characters in the two without-radical conditions. In the two with-radical conditions, the pseudo-characters were assigned meanings related to their semantic radicals. For example, the assigned meaning of 
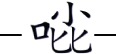
, (a special way of singing), was associated with the meaning of the radical 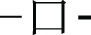. Having consulted the teachers and the students in a pilot study, the meaning assigned to each pseudo-character was ensured to be familiar to the participants. Pronunciations of the characters were randomly assigned in a way that no pseudo-characters included a phonetic component. In other words, the pronunciations of any of the pseudo-characters were not associated with any part of the characters.

##### Procedures

In order to accommodate the participants' class schedules, the experiments were conducted in four sessions where 12 pseudo-characters, three from each experimental condition, were introduced in each session. Each session consisted of two phases, a study phase and a test phase, as described earlier.

In order for all participants to receive the same instruction, instructions were pre-recorded by a native speaker of English using a SONY digital audio recorder and presented through PowerPoint presentation during the study phase. At the beginning of the PowerPoint presentation, participants were informed that the relationship between the pseudo-characters and their meanings would be the focus of the subsequent assessment. They were also provided with a practice question illustrating how they would be assessed after the study phase. During the study phase, each pseudo-character was introduced on one slide with a picture that represented the character's meaning, along with the audio narration providing the definition of the character. Each session in the study phase lasted for approximately 11 min. The test phase examined how well the participants could recall the meanings of the pseudo-characters they had just studied in a multiple-choice tasks. For each question, participants were asked to choose from four pictures the one that best represented the meaning of the pseudo-character.

#### Radical awareness

Radical awareness is a construct that consists of multiple facets: understandings of the forms of the radicals, the positional regularity, and their semantic category. Following Kuo et al. (2014), the present study focused on the positional regularities and the forms of radicals and used the same measure used in Kuo et al. (2014), which was adapted from the Chinese Orthography Choice task (Wang et al., 2005). The Chinese Orthographic Choice task involved two conditions: awareness of radical position and awareness of radical form. In Kuo et al. (2014), 10 items were selected from each condition, which yielded a total of 20 items on the measure. Each item was composed of two pseudo-characters, and the participants were asked to indicate which of the two was more likely to be a real Chinese character.

## Results

### Acquisition of characters varying in visual complexity and radical presence

To address the first research question, How two core properties of Chinese characters, visual complexity and radical presence, affect the acquisition of Chinese characters among adolescent beginning Chinese readers, data from the character acquisition task were analyzed in a 2 × 2 [Visual Complexity (more-stroke and fewer stroke) × Radical Presence (with-radical and without-radical)] repeated measure analysis of variance. Visual Complexity and Radical Presence were the within-participant variables. Table 2 presents the means and standard deviations of proportions correct on the character acquisition task.

**Table 2:** **Means and standard deviations of proportion correct on radical awareness and character acquisition measures**.

**Measures**	**Mean**	**SD**
Radical awareness	0.41	0.30
**CHARACTER ACQUISITION**		
**Few strokes**		
With-out radical	0.61	0.27
With-radical	0.71	0.18
**More strokes**		
With-out radical	0.55	0.19
With-radical	0.59	0.20

The hypothesis that the acquisition would be easier for characters with less visual complexity and with the presence of radicals was confirmed. The main effect of the radical presence was significant, *F*_(1, 22)_ = 7.08, *p* < 0.01, η^2^ = 0.24. Participants scored significantly higher on characters with radicals than characters without radicals. The main effect of visual complexity, as indicated by the number of strokes, was also statistically significant, *F*_(1, 22)_ = 9.93, *p* < 0.01, η^2^ = 0.31, with characters with fewer strokes being acquired significantly more easily than those with more strokes. The interaction between radical presence and visual complexity was not significant, *F*_(1, 22)_ = 1.11, *p* = 0.30.

### Relationship between radical awareness and character acquisition

To address the second research question, *How radical awareness is related to the acquisition of Chinese characters varying in visual complexity and radical presence*, correlational analysis was first performed among all measures. As shown in Table 3, the correlations were all significant, *p* < 0.05. The correlations among the character acquisition measures ranged from a moderate coefficient of 0.49 between the two with-radical conditions to a strong coefficient of 0.77 between the two without radical conditions.

**Table 3:** **Correlations between the measures**.

**Measures**	**1**	**2**	**3**	**4**	**5**
1. Fewer strokes_without radical	—				
2. Fewer strokes_with radical	0.729^**^	—			
3. More strokes_without radical	0.769^**^	0.711^**^	—		
4. More strokes_with radical	0.539^**^	0.494^*^	0.630^**^	—	
5. Radical awareness	0.523^*^	0.633^**^	0.424^*^	0.539^**^	—

** *The correlation was significant at the 0.01 level (2-tailed)*.

* *Correlation is significant at the 0.05 level (2-tailed)*.

The hypothesis that radical awareness would be associated with the acquisition of Chinese characters with radicals but not characters without radicals, regardless of visual complexity, was not confirmed. Correlational coefficients between the radical awareness and character acquisition measures ranged from 0.42 to 0.66. The correlations were slightly higher with the two with-radical conditions than those with the two without-radical conditions. Further analysis revealed that there was no statistical differences in the strength of correlations between the each condition and the radical awareness measure, $\chi_{\left(8\right)}^{2}=1.95$, *p* = 0.95, which may be due to the small sample size for this analysis.

To further examine the relationship between individual learner differences in radical awareness and the acquisition of characters varying in visual complexity and radical presence, data was reanalyzed in a 2 × 2 [Visual Complexity (more-stroke and fewer stroke) × Radical Presence (with-radical and without-radical)] mixed-design ANCOVA with radical awareness as the covariate. Visual Complexity and Radical Presence were the within-participant variables. The results showed that with radical awareness being considered, the effect of Radical Presence became non-significant, *p* = 0.14, and the effect of Visual Complexity became marginally significant, *p* = 0.04, η^2^ = 0.13.

## Discussion

### Acquisition of characters varying in visual complexity and radical presence

The present study shows that character properties have significant impact on the acquirability of the meaning of Chinese characters among beginning adolescent English-speaking learners of Chinese. First, in terms of visual complexity, the present study shows that characters with fewer strokes are generally easier to acquire than characters with more strokes. This finding is consistent with Kuo et al. (2014), which was conducted with young native-speaking beginning readers of Chinese. The finding is also largely consistent with reaction-time-based studies on the processing of Chinese characters, which showed that characters with fewer strokes are recognized more rapidly than characters with more strokes (e.g., Just and Carpenter, 1987; Tan and Peng, 1990; Su and Samuels, 2010). Drawing upon research on the visual complexity effect (e.g., Just and Carpenter, 1987; Tan and Peng, 1990; Su and Samuels, 2010), the greater acquirability of characters with fewer strokes observed in the present study can be attributed to a processing mechanism that encodes visual forms of words component by component. In other words, adult beginning learners of Chinese may have adopted a more analytical than holistic approach to processing unfamiliar Chinese characters and encoded unfamiliar characters stroke by stroke. Based on this encoding mechanism, the superior performance in the acquisition of characters with fewer strokes over those with more strokes can thus be explained in terms of limited working memory capacity: the more strokes a character has, the greater load it places on working memory, which limits the amount of working memory capacity available for associating a character with its corresponding meaning and for retaining such association.

It should be noted that the observed effect of visual complexity should not be generalized to the processing of high-frequency characters or characters learners are already familiar with. In the present study, the focus was more on the acquisition of the meaning of *new* characters and therefore all the stimuli used were novel characters. Hence, the observed effect of visual complexity may be limited to the processing of unfamiliar or low-frequency characters, but not familiar or high-frequency characters. Presence of visual complexity effect on the processing of only unfamiliar or low-frequency stimuli has been consistently documented in existing literature on reading speed literature (Jared and Seidenberg, 1990; Weekes, 1997; Ferrand, 2000; Juphard et al., 2004).

The observed effect of visual complexity with adolescent learners also complements findings from reaction-time-based research. Su and Samuels (2010) showed in a cross-sectional study that the effect of visual complexity on character judgment was present only among elementary school students, but not among middle school or university students. Note that Kuo et al. (2014) was also a cross-sectional study and involved children from grades 1 through 3, but no age-related decline in the effect of visual complexity in character processing was observed as revealed in Su and Samuels (2010). Since, we adopted the same experimental procedures used in Kuo et al. (2014), taken together, these findings may suggest a complex interaction among age, the effect of visual complexity and the aspect of character processing. Developmental differences in the effect of visual complexity may be more prominent in character judgment, as shown in Su and Samuels (2010), but not in character acquisition, as shown in Kuo et al. (2014) and in the present study. Confirming such speculation requires simultaneous investigation of both character judgment and character acquisition across learners of development age groups, which is beyond the scope of the present study but a points to promising direction for future research.

With regard to the second character property examined in the present study, radical presence, the findings corroborate those from reaction-time-based (Liu et al., 2010) and paper-based studies (e.g., Shu and Anderson, 1997; Ho et al., 1999, 2003a; Wu et al., 2009) and further demonstrate that the effect of radical presence on character acquisition extends to older beginning learners of Chinese. The adolescent second language learners of Chinese in our study shared similar character acquisition process with the young native-Chinese readers. Taking an analytical approach, our participants attended to the semantic radicals, and used radicals to infer and retain the meaning of new characters. This approach can be best explained by the Dual-Coding Theory (Sadoski and Paivio, 2013) from a verbal–non-verbal perspective. According to Dual Coding Theory, meaningful learning of characters occurs through the association of the novel characters with the verbal definitions and non-verbal pictures. The experiment focused on matching the characters with the corresponding picture, which is a measure of meaningful verbal-to-nonverbal referential processing. The use of both verbal and non-verbal codes as defined by Dual-Coding Theory thus plays a significant role in learning to recognize Chinese characters as well as to learning their meaning.

Somewhat interestingly, participants in our study had relatively low radical awareness as compared to the participants in Kuo et al. (2014). The means for the proportion correct on the radical awareness measure in Kuo et al. (2014) were around 90%; our study used the same radical awareness measure and the means for the proportion correct was around 50%. However, despite having more limited radical awareness, these adolescent second language learners of Chinese were able to decompose characters into informative semantic parts and utilize such knowledge in learning new characters.

In contrast to findings from Kuo et al. (2014), the present study did not find an interaction between visual complexity and radical presence. In Kuo et al. (2014), a significant joint effect of these two character property factors on meaning acquisition was revealed. More specifically, radical presence was found to have a significant effect on students' performance regardless of whether the pseudo-characters had greater or less visual complexity. The effect of visual complexity, however, was only significant for the characters without radicals, but not for those with radicals. These patterns were not detected in the present study with adolescent second language learners of Chinese. Taken together, these findings suggest that for young beginning learners of Chinese, characters with radicals are more acquirable than characters without radicals regardless of the number of strokes; characters with fewer strokes are more acquirable than those with more strokes only when the characters do not contain any radicals. However, for older beginning learners of Chinese, the effects of radical presence and visual complexity were independent. Such difference can be attributed to a combined effect of the differences in working memory and radical awareness between the participants in these two studies. Participants in Kuo et al. (2014) were younger and thus had smaller working memory capacity. However, they had more heightened radical awareness. Thus, when processing a character contains a radical, because of their relatively limited working memory, they were more likely to chunk the configurations of strokes into bigger components. Contrastively, in the present study, these older learners had greater working memory capacity but more limited radical awareness. Therefore, they were less likely to recognize the radicals but the greater working memory capacity allowed them to process the novel characters by strokes, which rendered the independence of these two factors in the present study.

### Relationship between radical awareness and character acquisition

Radical awareness was moderately correlated with the acquisition of all four types of characters. The significant correlations between radical awareness and the two without-radical conditions may appear somewhat unexpected at first glance. However, it should be noted that the pseudo-characters in the without-radical and the with-radical conditions shared the same stroke patterns and the only difference was that in the with-radical conditions, these stroke patterns served as radicals and contributed to the meaning of the characters whereas in the without-radical conditions, the same stroke patterns were positioned illegally as a radical and did not contribute to the meanings of the characters. Given that the radical awareness measure used in the present study focused on radical form and radical positions, the assessed radical awareness could have potentially contributed to the acquisition of the characters in the without-radical conditions. This interpretation is also in agreement with two observations. First, the correlations with radical awareness, while all significant, were higher with the two with-radical conditions than with the two without-radical conditions. Second, when the repeated measure analysis of variance was conducted with radical awareness as the covariate, the effect of radical presence became non-significant and the effect of visual complexity was weakened, despite being significant. Interestingly, although participants in the present study had much lower radical awareness, these observations are broadly consistent with findings in Kuo et al. (2014), which showed that for beginning readers, radical awareness contributed to the acquisition of characters with radicals but its relationship with the acquisition of characters without radicals was more tenuous.

## Limitations and future research

To our knowledge, the present study is the first to systematically examine the relationship between individual learner differences in radical awareness and the acquisition of characters varying in visual complexity and radical presence among non-native Chinese-speaking learners. This study has several limitations that warrant further investigation. First, as in Kuo et al. (2014), the scope of the present study is limited to factors that may contribute to the acquisition of the *meaning* of new characters, an area that has been consistently overlooked in research on visual processing. As noted earlier, the majority of the character reading research among beginning readers of Chinese has focused on character reading but not the acquisition of character meaning. However, because homophones are prevalent in Chinese, semantic aspects of character acquisition are likely to be more, if not equally, important than the phonetic aspects because successful phonetic decoding of a character does not always guarantee access to meaning (Anderson and Li, 2005). Nonetheless, we are fully aware of the multi-facetedness of the character acquisition process, which comprises semantic, orthographic (e.g., Wang and Geva, 2003) as well as phonetic aspects (e.g., Ho and Bryant, 1997; Hu and Catts, 1998; McBride-Chang and Ho, 2000; Kuo and Anderson, 2010; Luo et al., 2013). Future research should include tasks that involve all three aspects of character processing in a single study and investigate the effect of semantic, orthographic, and phonetic properties of characters on character acquisition.

Second, the present study only examined the relationship between character meaning acquisition and one individual difference variable, radical awareness. Given the visual complexity of Chinese characters, multiple visual processing skills have been studied in previous research on Chinese character acquisition, including visual perception (e.g., Ho and Bryant, 1999; Meng et al., 2002), visual spatial relationships (e.g., McBride-Chang et al., 2005b), visual discrimination (e.g., McBride-Chang et al., 2005b; Luo et al., 2013), visual closure (e.g., Chen and Kao, 2002; McBride-Chang et al., 2005b), visual sequential memory (e.g., Siok and Fletcher, 2001), visual paired associates (e.g., Huang and Hanley, 1995) and visual analogical skills (Kuo et al., 2014). Except for Kuo et al. (2014), the majority of these studies have focused on the phonetic, instead of the semantic, aspects of the visual forms of the characters when studying the relationship between visual skills and character processing. Since, Kuo et al. (2014) demonstrated how radical awareness and visual processing skills may interact to affect the acquisition of the meaning of characters varying in radical presence and visual complexity among young beginning native-Chinese readers, future research with non-native adolescent learners of Chinese should also be expanded to include individual differences in visual processing skills.

Third, the present study only focused on learners of Chinese who had prior literacy experience in English, an alphabetic language. As there with global increase in linguistic diversity (Xu, 2009), further research is needed on character acquisition that involved second language learners with prior literacy experience in languages varying in orthographies. Such cross-linguistic research would produce findings with important theoretical and practical implications.

Finally, the present study also highlights the need for instructional research on how Chinese characters can be more effectively taught to second language learners. Traditionally, Chinese characters are taught through pinyin and the emphasis is placed on the association of characters with phonetic information rather than the association with semantic information. Radicals, while introduced, were rarely a focus in initial Chinese-as-a-second-language instruction. The present study shows that radical awareness is significantly related to the acquisition of characters varying in properties even among beginning learners with limited radical awareness. Future intervention studies are warranted to investigate the effectiveness of instructional approaches with different emphases on character properties.

## Conclusion

The present study set out to address two important gaps in the existing literature on Chinese character processing. First, over the past two decades, research has focused on the effect of *either* visual complexity (e.g., Su and Samuels, 2010) *or* radical presence (e.g., Shu and Anderson, 1997; Perfetti and Tan, 1998; Feldman and Siok, 1999; Zhou et al., 1999; Perfetti et al., 2013) on character acquisition. To obtain a more comprehensive understanding of character acquisition, the present study draws upon theories of visual complexity effect (Su and Samuels, 2010) and dual coding processing (Sadoski and Paivio, 2013) and examined how visual complexity and radical presence can *jointly* affect the process. Second, the majority of the existing research on character acquisition has been conducted with young native-Chinese-speaking children. With the dramatic expansion of Chinese programs in K-12 in the U.S. and around the world, it is imperative to examine variables contributing to literacy development in Chinese from a broader cross-linguistic perspective. The present study, focusing on adolescent beginning English-speaking learners of Chinese, took the first step into this new research direction.

### Conflict of interest statement

The authors declare that the research was conducted in the absence of any commercial or financial relationships that could be construed as a potential conflict of interest.
